# A systematic review of diabetic foot infections: pathogenesis, diagnosis, and management strategies

**DOI:** 10.3389/fcdhc.2024.1393309

**Published:** 2024-08-06

**Authors:** Sabyasachi Maity, Noah Leton, Narendra Nayak, Ameet Jha, Nikhilesh Anand, Kamala Thompson, Danielle Boothe, Alexandra Cromer, Yaliana Garcia, Aliyah Al-Islam, Samal Nauhria

**Affiliations:** ^1^ Department of Physiology, Neuroscience, and Behavioral Sciences, St. George’s University School of Medicine, St. George’s, Grenada; ^2^ Department of Microbiology, St. Matthews University School of Medicine, Grand Cayman, Cayman Islands; ^3^ Department of Anatomy, St. Matthews University School of Medicine, Grand Cayman, Cayman Islands; ^4^ Department of Medical Education, School of Medicine, The University of Texas Rio Grande Valley, Edinburg, TX, United States; ^5^ Department of Pathology, St. Matthews University School of Medicine, Grand Cayman, Cayman Islands

**Keywords:** diabetic foot infection, antimicrobial resistance, podiatry, gram negative (G -) bacteria, gram positive (G +) bacteria

## Abstract

**Background:**

Diabetic foot infection represents a significant complication of diabetes mellitus, contributing substantially to morbidity, mortality, and healthcare expenditure worldwide. Accurate diagnosis relies on a comprehensive assessment integrating clinical evaluation, imaging studies, and microbiological analysis. Management necessitates a multidisciplinary approach, encompassing surgical intervention, antimicrobial therapy, and advanced wound care strategies. Preventive measures are paramount in reducing the incidence and severity, emphasizing patient education, regular foot screenings, and early intervention.

**Methods:**

The researchers performed a systematic review of literature using PUBMED MESH keywords. Additionally, the study was registered in the International Prospective Register of Systematic Reviews at the Center for Reviews and Dissemination, University of York (CRD42021277788). This review provides a comprehensive overview of the microbial spectrum and antibiotic susceptibility patterns observed in diabetic foot infections.

**Results:**

The search through the databases finally identified 13 articles with 2545 patients from 2021 to 2023. Overall, the predominant Gram-positive microbial species isolated were Staphylococcus aureus, Enterococcus fecalis, Streptococcus pyogenes, Streptococcus agalactiae, and Staphylococcus epidermidis. Whereas the predominant Gram-negative included Escherichia coli, Klebsiella pneumoniae, Proteus mirabilis and Pseudomonas aeruginosa.

**Conclusion:**

Diabetic foot infections represent a complex and multifaceted clinical entity, necessitating a holistic approach to diagnosis, management, and prevention. Limited high-quality research data on outcomes and the effectiveness of guideline recommendations pose challenges in updating and refining existing DFI management guidelines.

**Systematic review registration:**

https://www.crd.york.ac.uk/prospero/display_record.php?ID=CRD42021277788, identifier CRD42021277788.

## Introduction

Diabetic foot infections (DFIs) represent a complex and challenging complication of diabetes mellitus, presenting a significant burden on healthcare systems worldwide. These infections, primarily triggered by neuropathy and vascular complications associated with diabetes, often lead to severe consequences such as tissue damage, limb amputation, prolonged hospitalization, and increased mortality rates. Understanding the microbiological profile and antibiotic sensitivity patterns of organisms causing DFIs is crucial in guiding appropriate therapeutic interventions and improving clinical outcomes for affected individuals.

Roughly 18.6 million individuals worldwide experience diabetic foot ulcers annually, with 1.6 million cases reported in the United States alone. These ulcers precede 80% of lower extremity amputations in individuals diagnosed with diabetes and are correlated with heightened mortality rates (1). The pathophysiology of DFIs is intricately linked to the underlying microvascular and neuropathic complications of diabetes mellitus. Peripheral neuropathy, characterized by sensory loss and motor impairment, predisposes individuals to foot deformities and altered biomechanics, increasing the risk of pressure injuries and ulcer formation (2). Concurrent peripheral arterial disease exacerbates tissue ischemia, impairing wound healing and creating a favorable environment for infection (1). The interplay between these factors underscores the importance of preventive foot care strategies and early intervention to mitigate the risk of DFIs and their sequelae.

Microbiologically, DFIs encompass a diverse array of pathogens, with Staphylococcus aureus emerging as a predominant causative organism across various studies (3). However, the microbial profile of DFIs exhibits considerable heterogeneity, influenced by factors such as geographic location, patient demographics, and local antimicrobial resistance patterns. Recent research has highlighted the growing incidence of multidrug-resistant organisms, including Pseudomonas aeruginosa and MDR gram-negative bacilli, posing significant challenges for empirical antibiotic therapy (3). Understanding the microbial epidemiology of DFIs is essential for guiding antimicrobial stewardship efforts and optimizing treatment outcomes.

Clinically, the diagnosis of DFIs relies on a combination of clinical, biochemical, and radiographic findings to accurately assess the extent and severity of infection (4). While bone biopsy remains the gold standard for diagnosing osteomyelitis, its invasive nature and potential complications limit its routine use in clinical practice. Consequently, clinicians often rely on a combination of advanced imaging modalities, such as magnetic resonance imaging (MRI) and computed tomography (CT), alongside deep tissue cultures to guide therapeutic decisions effectively. Timely and accurate diagnosis is paramount to initiate appropriate management promptly and prevent further complications, including limb loss and systemic spread of infection.

Management of DFIs necessitates a multidisciplinary approach, encompassing surgical intervention, antimicrobial therapy, and comprehensive wound care to address the complex nature of these infections (4). Surgical debridement plays a pivotal role in source control and removal of necrotic tissue, particularly in cases of deep or severe infections. In osteomyelitis, surgical resection of infected bone may be curative, reducing the risk of recurrent infection and subsequent amputation. Antimicrobial therapy should be tailored to the individual patient and guided by culture and susceptibility testing to optimize outcomes and minimize the risk of antibiotic resistance. Additionally, comprehensive wound care, including off-loading strategies and advanced dressings, is essential for promoting wound healing and preventing recurrence.

Preventive measures play a crucial role in reducing the incidence and severity of DFIs, emphasizing the importance of patient education, regular foot assessments, and early intervention (2). Identifying high-risk individuals and implementing targeted interventions, such as diabetic foot care clinics and structured foot care programs, can significantly reduce the burden of DFIs and their associated complications. Furthermore, ongoing research efforts focusing on novel diagnostic modalities, antimicrobial therapies, and wound healing strategies hold promise for advancing the management of DFIs and improving clinical outcomes for individuals with diabetes-related foot complications.

Recent advancements in medical research have emphasized the need for a comprehensive evaluation of the microbial spectrum involved in DFIs and their susceptibility to various antibiotics. A myriad of studies, as evidenced by the systematic review conducted herein, have endeavored to elucidate the intricate relationship between diabetic foot infections and antimicrobial resistance patterns. This review synthesizes data from 13 pertinent articles obtained through meticulous searches across PubMed and other reputable databases, aiming to provide a consolidated perspective on the prevailing microbial flora in diabetic foot infections and the evolving trends in antibiotic susceptibility.

In this context, understanding the epidemiology, microbiology, and antibiotic resistance profiles of organisms causing DFIs is pivotal for optimizing therapeutic strategies, facilitating early intervention, and curtailing the escalating global burden of diabetic foot complications. This review article aims to critically analyze and synthesize the existing literature to offer a comprehensive overview of the microbial spectrum and antibiotic susceptibility patterns observed in diabetic foot infections, ultimately contributing to enhanced clinical management practices.

## Materials and methods

The researchers adhered to the Preferred Reporting Items for Systematic Reviews and Meta-Analyses (PRISMA) and Meta-Analysis of Observational Studies in Epidemiology (MOOSE) protocols (5, 6). Additionally, the study protocol was registered in the International Prospective Register of Systematic Reviews (PROSPERO) at the Center for Reviews and Dissemination, University of York (CRD42021277788), before the project commenced.

### Search strategy

Previous research has reviewed and summarized the antimicrobial resistance pattern in diabetic foot infection patients in South Asia between 2016 and 2021 (7). Therefore, published studies worldwide were searched in PubMed (US National Library of Medicine, National Institutes of Health) for potentially relevant studies from 2021 up to 2023. Articles published in English were included. The authors were required to reach a consensus among themselves on the final search strategy. The medical subject headings (MeSH) search terms included “Drug Resistance, Microbial,” “Diabetic Foot” and “Diabetes Mellitus” including all subheadings. The search strategy included (“Diabetic Foot”[Mesh]) AND “Drug Resistance, Microbial”[Mesh]. Finally, the relevant articles were also included by adopting the snowball method which involves searching the bibliographic list of selected articles.

### Selection of studies

Two independent reviewers (SN and NN) screened the retrieved papers based on titles and abstracts. Criteria for examination of full text of the relevant paper after the initial database screening were as follows:

Articles reporting data on Drug Resistance, Microbial,” “Diabetic Foot” and “Diabetes Mellitus that could be extracted for systematic review were included. Original studies conducted in any geographical location that provided a comprehensive overview of the microbial spectrum and antibiotic susceptibility patterns observed in diabetic foot infections were included in the final analysis.

The non-peer-reviewed editorials, letters, commentaries, incomplete data, reviews, conference posters, preprints, and thesis were excluded.

**Figure 1:**
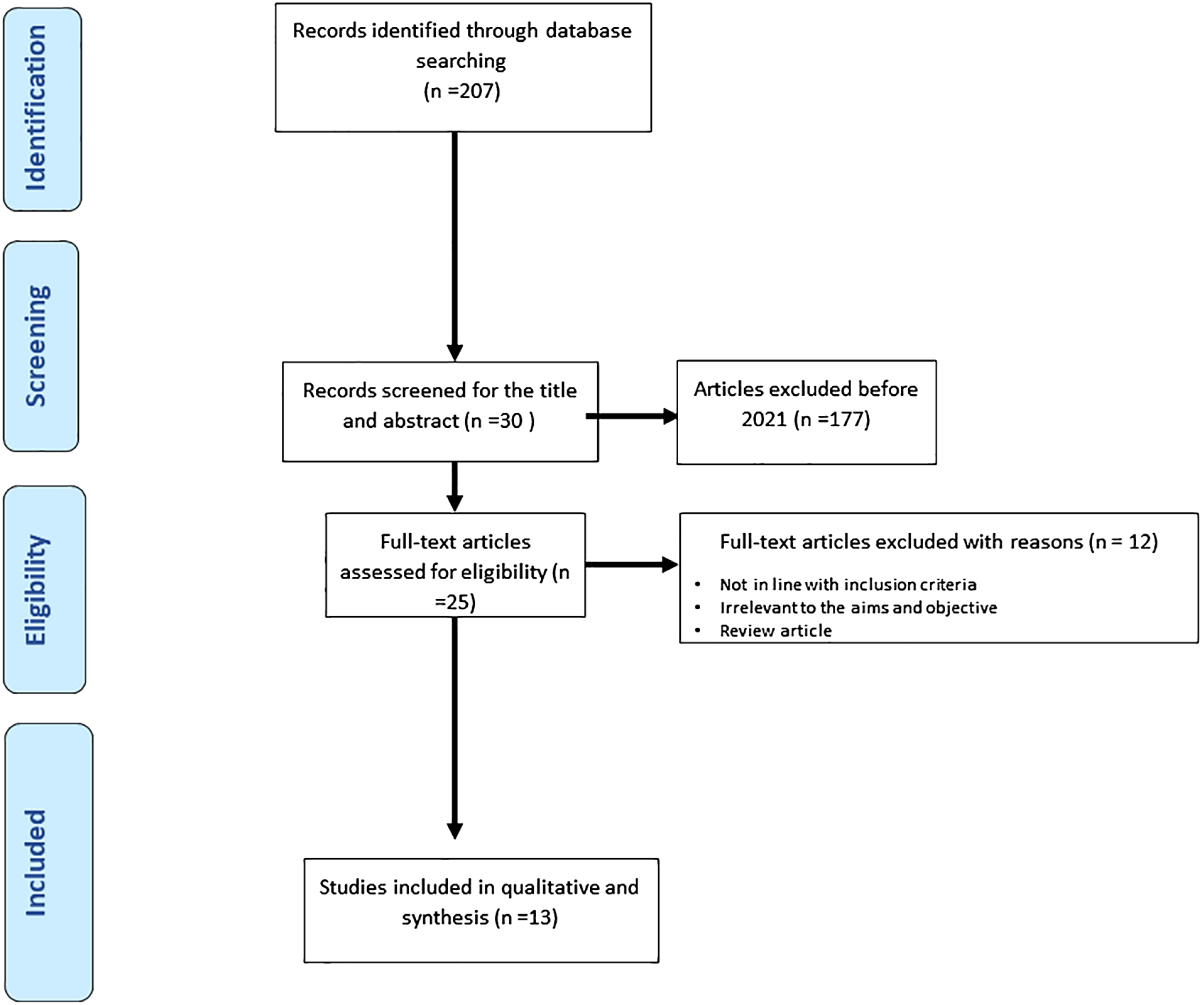
Represents the process of study selections for the systematic review as per the PRISMA protocol.

Any confusion or doubts regarding the study selection were resolved by reaching a consensus. The full PRISMA flow diagram outlining the study selection process is available in **Figure 1**.

### Data extraction

The authors extracted the relevant data, and the data was cross-checked. In a blank Excel sheet, data on year, authors, region, age range of patients, total included patients number, most prevalent pathogens, resistance pattern of Gram positive and Gram negative bacteria, and Study’s Focus were extracted.

### Quality assessment of included studies

The quality of the studies was assessed using the JBI Critical Appraisal Tool for Analytical cross sectional studies (8). All of the 13 included studies received at least 5 “YES” answers and were included in the systematic review synthesis (**Table 1**).

**Table 1:** Quality assessment of studies using JBI’s Critical Appraisal Tools designed for Analytical Studies.

Author/Year	Q1	Q2	Q3	Q4	Q5	Q6	Q7	Q8	Overall Score	Include
Fetni et al., 2023 (9)	Y	Y	U	Y	U	NA	Y	Y	5	Y
Taki et al., (10)	Y	Y	Y	Y	Y	NA	Y	Y	7	Y
Liu et al., (11)	Y	Y	Y	Y	U	NA	Y	Y	6	Y
Khaldi et al., (12)	Y	Y	Y	Y	N	NA	Y	U	5	Y
Atlaw et al., (13)	Y	Y	Y	Y	Y	NA	Y	U	6	Y
Permana et al., (14)	Y	Y	Y	Y	Y	NA	U	U	5	Y
Li et al., (15)	Y	Y	Y	Y	Y	NA	Y	Y	7	Y
Hung et al., (16)	Y	Y	Y	Y	Y	NA	Y	Y	7	Y
Saltoglu et al., (17)	Y	Y	Y	Y	Y	NA	Y	Y	7	Y
Savon et al., (18)	Y	Y	Y	Y	N	NA	Y	Y	6	Y
Siddiqui et al., (19)	Y	Y	Y	Y	N	NA	Y	Y	6	Y
Thanganadar Appapalam et al., (20)	Y	Y	Y	Y	U	NA	Y	Y	6	Y
Al-Mijalli, (21)	Y	Y	Y	Y	Y	NA	Y	Y	7	Y

## Results

### Search results and study characteristics

Our search through the databases finally identified 13 articles on diabetic foot infection and antibiotic resistance patterns that were included in the systematic review from 2021 to 2023. The article exclusion criteria included the following reasons:

Not relevant to the objective

Not in line with the inclusion criteria

The full-text pdf was not available

Not original research

No availability of statistical results

A detailed synthesis of included studies is provided in **Table 2**.

**Table 2:** A detailed qualitative synthesis of included studies.

Author/Year	Region	Age range (Years)	Total patients	Most prevalent pathogens	Sensitivity/ Resistance pattern Gram-positive bacteria	Most effective antibiotics Against Gram-negative bacteria	Conclusions
Fetni et al., (9)	Algeria, N. Africa	46 +/- 13	150	Gram negative bacilli- GNB (78.56%). E. coli (20%), Pseudomonas aeruginosa (15.33%)		E. coli treated with Fusidic acid (MR=1%), treated with Ofloxacin (2%), Ciprofloxacin (2%), Vancomycin (2%).	The diabetic foot (DF) is a major public health problem, with infection of the DF being a major risk factor for amputation. The multidisciplinary management of diabetic foot is still very precarious, and the best treatment is prevention.
Taki et al., (10)	Tehran	59.3 +/- 12.1	115	Staphylococcus spp. (52.2%), E. coli (33.3%)	Resistance to Clindamycin (73.5%, Ciprofloxacin (70.6%), Erythromycin (70.6%)	Cephalosporins, Ciprofloxacin	It is estimated that 10-25% of diabetic patients will encounter diabetic foot ulcers (DFU) during their lifetime. GPBs are the most common isolates from DFIs. Furthermore, with the development of wounds and infection, the prevalence of GNB in DFIs are increased.
Liu et al., (11)	South China	61.29 +/- 11.5	581	387(58.99%) - Gram Positive Organisms (GPO), Enterococcus, Staphylococcus (including MRSA)	Vancomycin, Linezolid, Tigecycline	Amikacin (97.14%), Meropenem (92%), Ertapenem (80%)	Most appropriate antimicrobial therapy should be selected based on pathogen culture and antimicrobial susceptibility.
Khaldi et al., (12)	Ouargla, Algeria	30-81	76	Proteus mirabilis (31), E. coli (13), Morganella morganii (11), Klebsiella pneumoniae (9)	Amoxicillin, amoxicillin-clavulanic acid, cephalexin, cefotaxime, ceftazidime, cefepime, aztreonam. 62.5% resistant to cefoxitin	Two Klebsiella pneuomoniae were carbapenem-resistant (ertapenem and imipenem)	Varying pathogens in DFI from one part of the country to another. This study reports the first description of metallo-B-lactamase NDM-5 producing Klebsiella pneumoniae clinical isolate in Algeria.
Atlaw et al., (13)	Addis Ababa, Ethiopia	54 ± 7	130	S. aureus (32/127-25.19%), Pseudomonas species (24/127-18.89%), E. coli (21/127-16.53%)	Gram positive sensitive to chloramphenicol, clindamycin, amikacin	Gram negative sensitive to chloramphenicol, aztreonam, amikacin	Importance of timely identification of infection of DFU, proper sample collection for identification of the pathogen and determining their antibiotic susceptibility pattern before initiating antimicrobial treatment.
Permana et al. (14)	Bandung, Indonesia	58 +/- 12.2	45	GNB (54 growth - 83.07%): Klebsiella pneumonia (13, 20%)	Tigecycline (100%), Vancomycin (100%), Gentamycin (90.9%, Meropenem (100%), Ertapenem (100%),	Carbapenems (meropenem and ertapenem) except for Acinetobacter baumanii, amikacin (96.2%)	No susceptibility pattern difference between patients with ulcer duration less than or greater than 2 months, higher grade (Wagner 4 and 5) and lower, as well as patients with previous or no antibiotic history.
Li et al., (15)	Chongqing, China	60.32 +/- 11.62	101	S. aureus, Enterococcus faecalis and Streptococcus agalactiae. Most common GNB: Pseudomonas aeruginosa, Klebsiella pneumoniae, Proteus mirabilis	Levofloxacin (90.0%, Moxifloxacin (90.0), Vancomycin (100%), Teicoplanin (100%), Tigecycline (100%), Linezolid (100%).	Amikacin (93.3%), Piperacillin/tazobactam (93.9%), Cefoperazone/sulbactam (92.9%), Ceftazidime, Cefepime, Imipenem (100%), Meropenem (100%).	Diabetic foot ulcers complicated by necrotizing fasciitis (DNF). Investigated the distribution and susceptibility of pathogenic bacteria in DNF patients, and provided empirical antibacterial guidance for the clinic.
Hung et al., (16)	Taiwan	62.9 +/- 14.0	558	Streptococcus spp. (25.8%), methicillin-sensitive Staphylococcus aureus (21.7%), methicillin-resistant Staphylococcus aureus (12.5%). GNB: Proteus spp. (16.3%), E. coli (11.1%), Pseudomonas sp. (8.8%).	Glycopeptide against methicillin-resistant Staphylococcus aureus (MRSA) was also prescribed in 26% of patients.	Broad-spectrum antibiotics against gram-negative and anaerobic pathogens, including third-generation cephalosporin (43%), extended-spectrum penicillin (31% with aminopenicillins and 5% with ureidopenicillins), fluoroquinolones (6%), carbapenems (5%), and metronidazole (19%) were prescribed promptly for these patients initially.	Association between specific bacterial pathogens and treatment outcomes in patients with limb-threatening diabetic foot infections (LT-DFI) were investigated. The presence of GNB was associated with limb amputations. This study provides insight into more timely and appropriate management.
Saltoglu et al., (17)	Turkey	59.9 +/- 11.3	284	S. aureus (36, 14.6%), E. coli (32, 13.0%), Methicillin resistant (19.4%) S. aureus and coagulase-negative (69.9%) Staphylococcus spp., Multidrug-resistant Pseudomonas aeruginosa in 4 of 22(18.2%), Extended-spectrum beta-lactamase-producing Gram-negative bacteria (38.5%)	Beta-lactam/beta-lactamase inhibitor combinations (74%), Piperacillin/tazobactam (56.9%), Amoxicillin/clavulanate (27.0%), Daptomycin (18.0%)		Importance of using appropriate narrow-spectrum empirical antimicrobials because higher rates of reinfection and major amputation were found in use of broad-spectrum antimicrobials.
Savon et al., (18)	Zaporizhzhya, Ukraine	56.8 +/- 2.5	210	Gram-positive flora in 118(56.2%), Gram-negative in 81(38.6%). S. aureus in 52(24.7%); MRSA in 38(73.0%). Enterococcus faecalis in 29(13.8%). Pseudomonas aeruginosa in 23(10.9%).	3rd and 4th generation Cephalosporins - no resistance detected. No phenotype of resistance was detected in Linezolid, Trimethoprim/Sulfamethoxazole, and all drugs from the group of tetracyclines. Sensitivity level of more than 92% was found in Amikacin, Gentamicin, Netilmicin, and moxifloxacin. A sensitivity level of up to 80% was found in Vancomycin, Teicoplanin.		We have identified a pattern that allowed us to categorize patients with MRSA into four groups according to similar sensitivity to antibacterial drugs, which received the conventional designations MRSA type 1; MRSA type 2; MRSA type 3; MRSA type 4. Moreover, MRSA type 4-3(7,9%) is pan-resistant.
Siddiqui et al., (19)		53.05 +/- 10.70	201	Gram-positive 151(38.76%) ; S. aureus 97(24.93%), Strep. Pyogenes 20(5.14%), Enterococcus spp. 28(7.19%). Gram-negative 238(61.24%); Proteus mirablis 58(14.91%), Pseudomonas aeruginosa 47(12.1%), E. coli 41(10.53%), Klebsiella pneumoniae 32(8.22%)	Gram positive organisms 100% sensitive to Vancomycin. Staph 100% sensitive to Linezolid. MRSA susceptibility pattern: Vancomycin 100%, Fucidic acid 75%, and Clindamycin 55%.	Gram negative organisms 100% sensitive to Colistin. Among 61.19% of gram-negative infections, proteus (14.91%) and pseudomonas (12.1%) accounted for majority of the isolates; Amikacin sensitivity was 74.14% and 76.19 respectively.	Early identification
Thanganadar Appapalam et al., (20)	Chennai, India	53.04 +/- 12.12	50	Gram-negative aerobes (66%); P aeruginosa (23.2%). Gram-positive isolates (34%)	All gram-positive isolates displayed resistance against penicillin and vancomycin. Insensitivity of amoxicillin to staphylococcus aureus, B subtilis, and streptococcus dysgalactiae and cefotaximine resistance to streptococcus dysgalactiae was noted. Amikacin was not more than 50% effective. All the gram-positive isolates displayed 100% sensitivity against gentamycin.	Majority of the gram-negative isolates showed (80-100%) sensitivity to cefotaxime.	Need for the discovery of novel drug(s) to alleviate antibiotic-resistance bacterial infections in DFU patients
Al-Mijalli, (21)	Saudi Arabia, Riyadh	59.4 +/- 7.6	44	Gram-negative 28(63.6%). Gram-positive 16(36.3%). S. aureus 29 (65.9%)	Stapylococcus aureus was most sensitive to Ciprofloxacin.	Most of the organisms were susceptible to Vancomycin, Ciprofloxacin, Cefalexin, but resistant to Methicillin, Gentamicin, Ampicillin.	The study concludes that While Vancomycin should be used empirically for Gram-positive isolates, Ciprofloxacin can be taken into consideration for most of the Gram-negatives aerobes. Based on including various microorganisms and the advent of multidrug-resistant strains, proper culture and sensitivity testing are necessary prior to the empirical therapy.

The microbiological Profile of Diabetic Foot Infections and the antibiotic resistance patterns were explored from the included studies. Overall, the predominant Gram-positive microbial species isolated in DFIs were Staphylococcus aureus, Enterococcus fecalis, Streptococcus pyogenes, Streptococcus agalactiae, and Staphylococcus epidermidis. Whereas the predominant Gram-negative included Escherichia coli, Klebsiella pneumoniae, Proteus mirabilis, Pseudomonas aeruginosa.

The antibiotic resistance patterns for the most common Gram-positive and Gram-negative species are listed in **Table 3**.

**Table 3:** Shows the antibiotic resistance patterns for the most common Gram-positive and Gram-negative species.

Organism	Studies	Penicillin	Amoxycillin-Clavulanic acid	Amoxycillin	Ciprofloxacin	Ofloxacin	Gentamicin	Amikacin	Erythromycin	Vancomycin	3rd gen cephalo- cefoperzone, cefotaxim, ceftazidime	Daptomycin	Clindamycin
Staph aureus	(9)	11%			6%	5%	11%		8%	0%			9%
	(10)				70.60%		11.80%		70.60%				73.50%
	(11)	95.62%			28.57%		8.03%		48.91%	2.19%			48.91%
	(13)	100%			50%		78%	18%	68.80%	37%			38%
	(14)		16.70%		33.30%		16.70%		20%	0%	Ct-16.7%, Cz-16.7%, Cx-16.7%		20%
	(15)	95%			17.6%		15%		65%	0%		0%	65%
	(17)												
	(18)	20-60%	0%	20-60%	20-60%		0%	0%		20-60%	Cz-100, Cx-0	20-60%	20-60%
	(19)							14.29%		0%			38.95%
	(20)	100%		100%	45%			82%		100%	Ct-82%		
Staph epid	(9)	11%											
	(11)	100.00%			100%		27.27%		81.82%	3.03%			66.67%
Strepto Pyo	(11)	10%			40%				80%	0%			72%
	(13)	100%			100%		100%		100%	100%			100%
	(19)									0%			40%
Strepto agalact	(15)	0%			33.3				83.3	0%		0%	77.8
Entero fecalis	(9)	10%		12%									
	(10)	41.90%								16.10%			
	(11)	4.62%			23.21%		2.86%		98.53%	4.48%			
	(13)	100%			75%		100%	100%	100%	50%			75%
	(15)	5.6			31.3		44.4		94.40%	0%		0%	100%
	(19)									0			
	(20)	100		20				40		100			

## Discussion

DFIs represent a complex and multifaceted clinical entity, necessitating a holistic approach to diagnosis, management, and prevention. By integrating existing research findings with insights from clinical practice, this review provides a comprehensive overview of DFIs, highlighting the challenges and opportunities for optimizing patient care and reducing the burden of this debilitating complication of diabetes mellitus. We set out to explore the antimicrobial resistance patterns amongst the patients of DFIs worldwide, and our results indicate the most prevalent bacteria. The most common Gram-positive and Gram-negative bacteria associated with DFI were Staphylococcus aureus and Escherichia coli, respectively.

### Pathogenesis and diagnosis of DFIs

The pathogenesis of DFIs is multifactorial, primarily driven by the underlying complications of diabetes, including peripheral neuropathy, peripheral arterial disease, and impaired immune function. Peripheral neuropathy leads to sensory loss and motor abnormalities, increasing the risk of unnoticed trauma and skin breakdown. Vascular compromise further impairs wound healing and predisposes individuals to infections. The presence of high blood glucose levels in diabetes also promotes bacterial growth and impairs immune responses, creating an environment conducive to infection development. These factors collectively contribute to the high incidence of DFIs in individuals with diabetes (4).

Diagnosing diabetic foot infections involves a thorough clinical assessment, including evaluating signs of inflammation, such as erythema, warmth, swelling, and purulent discharge. Diagnostic imaging modalities like X-rays and advanced imaging techniques may be utilized to assess for soft tissue involvement and osteomyelitis. Laboratory tests, such as elevated inflammatory markers like C-reactive protein and erythrocyte sedimentation rate, can aid in confirming the presence of infection. Additionally, microbiological cultures of wound samples help identify the causative pathogens, guiding appropriate antibiotic therapy. A multidisciplinary approach involving healthcare professionals specializing in wound care, infectious diseases, and podiatry is essential for the accurate diagnosis and effective management of diabetic foot infections to prevent complications and improve patient outcomes (4).

In recent years, advanced diagnostic techniques have been increasingly utilized for challenging cases of diabetic foot infections (DFIs) to improve accuracy and guide appropriate management. Molecular diagnostics, such as polymerase chain reaction (PCR) and next-generation sequencing (NGS), allow for the rapid and precise identification of microbial pathogens in DFIs. These molecular methods can detect a wide range of bacteria, including fastidious and anaerobic organisms, providing valuable information for targeted antimicrobial therapy (22). Advanced imaging techniques, such as magnetic resonance imaging (MRI) with contrast enhancement and positron emission tomography-computed tomography (PET-CT) scans, offer enhanced sensitivity and specificity in detecting soft tissue infections, osteomyelitis, and deep-seated abscesses. These modalities help in the accurate localization of infections and assessment of treatment response (23). Additionally, point-of-care testing, which includes rapid diagnostic tests for detecting specific pathogens or antibiotic resistance genes, is being developed to facilitate prompt decision-making regarding antimicrobial therapy. These tests enable healthcare providers to tailor treatment regimens based on the individual’s infection profile, leading to more effective outcomes (24). By incorporating these advanced diagnostic techniques into clinical practice, healthcare professionals can achieve earlier and more precise diagnoses of challenging cases of DFIs, leading to targeted and personalized treatment strategies that optimize patient care and outcomes.

### Antimicrobial resistance in DFIs

Analyzing two decades of research on antimicrobial resistance reveals a remarkable 450% surge in research activity from 1999 to 2018, with over 150,000 articles originating from 166 countries (25). Antibiotic resistance patterns and various resistance trends have been observed among members of Enterobacteriaceae, Pseudomonas aeruginosa, Staphylococcus aureus, and Enterococcus faecalis both temporally and globally. Among Enterobacteriaceae, the production of extended-spectrum beta-lactamases stands out as a common resistance mechanism (26). Notably, for Pseudomonas aeruginosa, there has been a significant decreasing trend in resistance rates to ciprofloxacin, ceftazidime, meropenem, and imipenem. This decline in resistance rates could potentially be attributed to reduced usage of ciprofloxacin among Pseudomonas aeruginosa isolates (27). Several factors can explain the decreased resistance of Pseudomonas aeruginosa to meropenem and imipenem. Changes in antibiotic utilization within healthcare settings can significantly impact the development of resistance. A shift towards more judicious prescribing of meropenem and imipenem may have decreased resistance rates over time (28). Furthermore, implementing antibiotic stewardship programs, which aim to optimize antibiotic use, promote appropriate prescribing practices, and prevent the emergence of resistance, likely play a crucial role in this observed trend (29). The evolution of bacterial strains is another important factor. P. aeruginosa is known for its rapid adaptation and evolution. Strains with lower intrinsic resistance to meropenem and imipenem may have become more prevalent, leading to an overall decrease in resistance rates (30). Additionally, environmental factors, such as changes in healthcare environments or exposure to different antimicrobial agents, can also influence the resistance patterns of P. aeruginosa over time (31).

Several antibiotics approved for treating various infections demonstrate potential efficacy in managing DFIs. For DFIs caused by Staphylococcus aureus, ceftaroline, dalbavancin, oritavancin, and tedizolid show promise. In cases of DFIs due to Pseudomonas aeruginosa and Enterobacteriaceae, several combination therapies have demonstrated effectiveness. These combinations include ceftazidime/avibactam, ceftolozane/tazobactam, imipenem/cilastatin/relebactam, and cefiderocol. Additionally, meropenem/vaborbactam and plazomicin can be utilized for infections caused by Enterobacteriaceae (32).

Diabetic foot infection stands as a primary contributor to non-traumatic lower limb amputations. However, pertinent data are scarce, and there is a notable absence of randomized controlled trials assessing the efficacy of these agents in this domain. Pending the availability of more substantial evidence, cefiderocol and dalbavancin, having undergone more comprehensive examination in patients with bone infections, could present appealing choices for carefully chosen individuals with severe diabetic foot infection (32). Several factors have been identified as risk factors in the development of antimicrobial resistance in DFI. These include high BMI, high glycosylated hemoglobin, elevated fasting blood glucose, course and size of the ulcer, peripheral neuropathy, and vascular disease. Also, compromise of the host’s immune system due to a decrease in leukocyte count and neutrophil ratio was identified (33). Most common bacteria in DFIs are Staphylococcus aureus, Escherichia coli, Pseudomonas aeruginosa, Proteus vulgaris, and Morganella morganii, all of which demonstrated antibiotic resistance to various medications including Ampicillin, Ciprofloxacin, Levofloxacin, Trimethoprim-sulfamethoxazole, and Cefuroxime. Among these antibiotics, Escherichia coli showed the most resistance (34). Even as gram negative bacteria have been implicated as the most common cause of DFIs, the most notable risk factors are hypertension and neuropathy. Also, the overuse of antibiotics has been found to play a role in the development of these drug resistant infections (35). These gram-negative bacteria were also isolated from patients with foot ulcers that have had an amputation. The most notable being Escherichia coli (36).

A study examined diabetic foot osteomyelitis and identified both gram positive and gram negative pathogens in bone cultures. These organisms included coagulase negative Staphylococcus, Staphylococcus aureus, Proteus species, Pseudomonas aeruginosa, and Escherichia coli. Although cultures were mostly polymicrobial, they were either gram-positive dominated or gram negative dominated. Penicillin without β-lactamase resistance was found in both cases, but sulphonamides were peculiar to gram negative dominated (37). In another study done in sub-Saharan Africa, Staphylococcus aureus showed the highest pooled resistance toward gentamicin and ciprofloxacin. E. coli and Klebsiella pneumoniae showed significant resistance rates for several common antibiotics (38).

In the case of polymicrobial infections in DFI, determining the specific species responsible for the infection can be challenging due to multiple microorganisms. Several factors contribute to the complexity of identifying the causative agent in polymicrobial infections. Firstly, synergistic interactions among microbial species can lead to enhanced virulence or antibiotic resistance, making it difficult to isolate and attribute the infection to a single organism (39). Additionally, the microbial composition of polymicrobial infections can vary between individuals and even within the same individual over time, complicating the identification of the primary pathogen responsible for the infection (40). Furthermore, conventional diagnostic methods may not always accurately identify all microorganisms present in a polymicrobial infection. Some organisms may be fastidious or difficult to culture, leading to underestimating their role in the infection (41). Lastly, many microorganisms in polymicrobial infections can form biofilms, protecting against antibiotics and host immune responses. Biofilms can consist of multiple species, further complicating the identification of the dominant pathogen (42). Host factors, including immune status, comorbidities, and anatomical factors, can also influence the microbial composition of polymicrobial infections. The host response to infection may also impact the relative abundance and pathogenicity of different microorganisms (43).

Given the challenges in identifying the responsible species in polymicrobial infections, a holistic approach to managing such infections is crucial. Comprehensive microbial analysis techniques, such as next-generation sequencing or metagenomic approaches, can offer a more detailed understanding of the microbial community dynamics. By acknowledging these complexities and limitations, researchers and clinicians can develop more effective treatment strategies that address the diverse microbial populations in polymicrobial infections.

### Risk factors for DFIs

The observed high antibiotic resistance risk associated with factors such as high BMI, elevated HbA1c levels, elevated fasting blood glucose, and the course and size of the ulcer in DFIs underscores the intricate relationship between host characteristics and microbial susceptibility to antibiotics. High BMI is known to be associated with chronic inflammation and impaired immune function, creating a favorable environment for microbial proliferation and antibiotic resistance. The increased adipose tissue in individuals with high BMI can serve as a reservoir for pathogens, leading to persistent infections and reduced antibiotic efficacy (33). Elevated HbA1c levels and fasting blood glucose contribute to the impaired immune response and delayed wound healing commonly observed in individuals with poorly controlled diabetes. The hyperglycemic environment promotes bacterial growth and biofilm formation, enhancing antibiotic resistance (33). The course and size of the ulcer in DFIs indicate the severity and chronicity of the infection. Larger and more chronic ulcers often harbor diverse microbial populations, including antibiotic-resistant strains. The presence of biofilms in chronic ulcers further complicates treatment and contributes to antibiotic resistance (42).

Host factors, including immune status, comorbidities, and anatomical factors, can influence the microbial composition of polymicrobial infections. The host response to infection may also impact the relative abundance and pathogenicity of different microorganisms (43). By elucidating the association between these host-related factors and antibiotic resistance in DFIs, the current review highlights the importance of comprehensive management strategies that address not only the microbial aspect but also the host factors influencing treatment outcomes. Future research focusing on personalized approaches considering individual patient characteristics and tailored antibiotic regimens may offer insights into mitigating the impact of antibiotic resistance in DFIs.

Recent research on DFIs highlights risk factors such as previous hospitalization, ulcer size, surgical therapy, and C-reactive protein. Regarding previous hospitalization, nosocomial infections are well-established culprits and arise due to the poor state of the wards and faulty antiinfection policies and procedures. For ulcers, ulcer size is an important prognostic factor in diabetic foot. An ulcer size >4cm^2^ is said to be a significant risk factor for DFIs. As regards surgical therapy (like amputation), the exact way it increases the risk of MDR remains unclear. A unique feature of surgical therapy is that it alters the biomechanics of the foot. For C-reactive protein, its increased level during bacterial infections is a risk factor for MDR DFI (11). Among infections severely endangering the affected limb, cultures most commonly identify pathogens such as Staphylococcus aureus, Enterococcus, facultative gram-negative bacilli, and group B streptococci; with the unhygienic nature of the hospital wards responsible for exaggerating the infections (44). Apart from previous history of antimicrobial exposure, wounds due to neurovascular defects, Wagner grade 3–5, and concurrent osteomyelitis are risk factors for AMR (45). Considering the high prevalence of antibiotic resistance in Escherichia coli and Klebsiella pneumoniae towards most antibiotics, policies, processes, and procedures must be implemented to ensure good hygiene and infection control to mitigate or eliminate the spread of these organisms (38).

The prevalence of AMR changed with onset of the Covid-19 pandemic, with a 3-fold increase in risk from the pre-pandemic period. Some factors responsible in studied population were antibiotic self-administration, prior hospitalization, and antibiotic prescription by general practitioners (46). In a hospital in Nicaragua, previous antibiotic usage was identified as a key contributor to the high prevalence of MRSA as well as the resistance rate exhibited by gram-negative organisms to various classes of antibiotics (47). Contrary to the above studies, another study revealed that the administration of antibiotics to patients with diabetic foot osteomyelitis up to a week prior to biopsy does not affect the culture result. Furthermore, such administration does not result in increased antibiotic resistance (48). Also, although recurrent episodes of DFI are likely to follow a successfully treated episode, the treatment does not increase the likelihood of AMR in these episodes (49).

### Clinical implications and treatment strategies

The MDT approach has proven effective in reducing DFUs and LEAs, yet there exists variability among team members and interventions. Podiatrists are proposed as pivotal in DFU prevention and management. A recent systematic review assessing podiatric interventions in MDTs for DFUs and LEAs, has emphasized the need for intervention clarity and role delineation in practice and literature (50). Another systematic review including thirty-three studies, aimed to evaluate how multidisciplinary teams impact major amputation rates among DFI patients. The MDT was structured to include a blend of medical and surgical disciplines, ensuring a comprehensive approach to diabetic foot ulcer management. Larger teams found organizational benefits by designating a “captain” and establishing a core team structure supplemented by ancillary members. This setup facilitated clear referral pathways and streamlined care algorithms, enabling timely and thorough management of diabetic foot ulcers. Multidisciplinary teams addressed a range of key tasks, including glycemic control, local wound management, vascular disease, and infection, ensuring a holistic approach to patient care. Notably, 94% of studies reported reduced major amputation rates with multidisciplinary teams, highlighting their effectiveness in addressing key aspects of diabetic foot care (51).

In 2019, led by the Jiangsu Medical Association and the Diabetes Society of the Chinese Medical Association, a writing group was convened for the development of the ‘Guidelines on Multidisciplinary Approaches for the Prevention and Management of Diabetic Foot Disease (2020 edition)’. These guidelines enlisted contributions from experts spanning endocrinology, burn injury, vascular surgery, orthopedics, foot and ankle surgery, and cardiology. They stress the criticality of timely wound assessment, diagnosis, and appropriate surgical interventions, both internally and externally, in managing diabetic foot pathology. The article strongly advocates for the establishment of multidisciplinary diabetic foot teams and specialist centers at various levels, underlining the urgency of prompt consultation or referral to these teams or centers based on the severity of the patient’s condition (52). A retrospective study on a multidisciplinary team led by internists revealed promising outcomes in diabetic foot ulceration management. The study encompassed 315 patients, with 207 treated during the pre-multidisciplinary period and 108 during the multidisciplinary period. Significant reductions in major amputations and bloodstream infections were observed during the multidisciplinary period compared to the pre-multidisciplinary phase (10% vs. 14%; p = 0.01 and 2% vs. 13%, p = 0.04, respectively). Moreover, there was a notable decrease in 30-day mortality rates (5% vs. 11%, p = 0.08) and a substantial increase in vascular interventions (18% vs. 1%, p < 0.01). Improvements in diabetes control were evident, with lower median glucose levels recorded (163 vs. 185 mg/dl, p = 0.03). Treatment modifications, including updates to medications such as angiotensin-converting enzyme inhibitors/angiotensin II receptor blockers and statins, were implemented alongside enhanced disease control indicated by improved laboratory results at discharge, including albumin and CRP levels (53).

### Challenges in developing and adopting guidelines for DFI management

The development and adoption of guidelines for the proper management of DFIs on a global scale face several hindrances that impede their effectiveness and implementation. Some of the key challenges include variability in healthcare systems and resources across different regions, which poses a challenge in standardizing guidelines for DFI management. Disparities in access to healthcare facilities, diagnostic tools, and antimicrobial agents hinder the uniform implementation of guidelines (54). Insufficient awareness among healthcare providers, patients, and caregivers about the importance of following guidelines for DFI management can lead to suboptimal adherence. Additionally, inadequate education and training programs on evidence-based practices contribute to the underutilization of guidelines (54).

Resource-constrained settings, especially in low- and middle-income countries, face challenges in implementing comprehensive DFI management guidelines due to limited infrastructure, lack of essential medications, and inadequate funding for healthcare services. Also, the complexity and length of guidelines for DFI management may deter healthcare providers from incorporating them into routine clinical practice. Guidelines that are overly detailed or difficult to interpret can lead to confusion and non-compliance (55). Effective management of DFIs often requires a multidisciplinary approach involving podiatrists, infectious disease specialists, endocrinologists, and wound care nurses. The lack of collaboration and communication among different healthcare professionals can hinder the implementation of guideline recommendations (54). Limited high-quality research data on DFI management outcomes and the effectiveness of guideline recommendations pose challenges in updating and refining existing guidelines. The lack of robust evidence-based practices can hinder the development of comprehensive and up-to-date guidelines (55).

Addressing these hindrances through targeted strategies such as capacity building, educational initiatives, simplified guideline formats, enhanced collaboration among healthcare professionals, and increased research efforts can help overcome barriers to the development and adoption of guidelines for the proper management of DFIs on a global scale (54, 55).

### Future perspectives and recommendations

Despite significant advancements in the management of DFIs, several gaps in current knowledge and research limitations persist, hindering optimal patient care and outcomes. One notable limitation is the lack of evidence-based guidelines guiding the choice and efficacy of topical treatments for DFIs. The choice of topical approaches, whether alone or as adjuncts to systemic antibiotics, is often not evidence-based due to a paucity of robust clinical trials (56). This gap underscores the need for well-designed studies to evaluate the efficacy and comparative effectiveness of various topical agents, including antibiotic-impregnated biomaterials, novel antimicrobial peptides, and photodynamic therapy. Furthermore, research emphasizes the need for clear evidence-based guidelines on the use of topical treatments in DFI management (57). Despite the increasing prevalence of antibiotic resistance, there is a lack of consensus on the optimal topical interventions to limit the use of systemic antibiotics and prevent disease progression. This highlights the urgent need for well-designed clinical trials to assess the efficacy, safety, and cost-effectiveness of emerging topical therapies. Another significant gap in current research lies in the understanding of antimicrobial resistance patterns and their impact on clinical outcomes in patients with DFIs. Previous research highlights the prevalence of multidrug-resistant organisms (MDROs) in DFIs, including methicillin-resistant Staphylococcus aureus (MRSA) and extended-spectrum beta-lactamase (ESBL)-producing Enterobacteriaceae (17). However, the study also underscores the need for appropriate empirical antimicrobial therapy, as higher rates of reinfection and major amputation were found even with the use of broad-spectrum antimicrobials. This emphasizes the importance of tailored antibiotic stewardship programs to optimize treatment outcomes while minimizing the risk of antimicrobial resistance.

Future research directions in understanding and combating antibiotic resistance in diabetic foot infections (DFIs) should focus on several key areas to address current challenges and optimize treatment outcomes. Firstly, there is a critical need for further investigation into the mechanisms of antimicrobial resistance in DFIs, including the identification of novel resistance mechanisms and the factors contributing to the emergence and spread of multidrug-resistant organisms (MDROs). Understanding the antimicrobial resistance patterns of pathogens is crucial for guiding empirical antibiotic therapy and minimizing treatment failures (17). Secondly, future research should focus on the development and evaluation of novel antimicrobial agents and treatment modalities for DFIs. This includes exploring alternative therapeutic approaches such as antimicrobial peptides, growth factors, and nanomedicine (58). Novel treatment modalities with broad-spectrum activity and low potential for inducing resistance could offer promising alternatives to conventional antibiotics and help mitigate the spread of antimicrobial resistance. Additionally, there is a need for well-designed clinical trials to evaluate the efficacy, safety, and cost-effectiveness of emerging antimicrobial therapies in DFIs. Comparative effectiveness studies comparing different treatment modalities and assessing long-term outcomes, such as wound healing, infection recurrence, and amputation rates, are essential for guiding clinical decision-making and optimizing patient care. Furthermore, future research should explore innovative strategies for antibiotic stewardship and infection control in DFIs. This includes implementing antimicrobial stewardship programs in healthcare settings, promoting judicious antibiotic use, and optimizing infection prevention and control practices to minimize the risk of antimicrobial resistance and healthcare-associated infections.

Effective strategies to mitigate antibiotic resistance in diabetic foot infections (DFIs) require a multifaceted approach involving both clinical practice and public health policies. Based on the current evidence and research findings, several recommendations can be made to address this pressing issue. Firstly, healthcare providers should prioritize judicious antibiotic prescribing practices in the management of DFIs. Inappropriate initial antibiotic treatment is associated with higher rates of reinfection and major amputation. Therefore, clinicians should adhere to evidence-based guidelines and perform comprehensive microbiological evaluations to guide empirical antibiotic therapy and minimize the risk of antimicrobial resistance. Furthermore, healthcare facilities should implement antimicrobial stewardship programs to promote responsible antibiotic use and optimize treatment outcomes in DFIs. These programs should involve multidisciplinary teams, including infectious disease specialists, microbiologists, pharmacists, and infection control practitioners, to develop and implement evidence-based guidelines, educate healthcare providers, and monitor antibiotic prescribing practices. In addition to clinical practice, public health policies are crucial in mitigating antibiotic resistance in DFIs. Governments and healthcare authorities should prioritize investments in the research and development of novel antimicrobial agents, as well as promote initiatives to incentivize antibiotic research and development, such as push funding mechanisms (56). Moreover, efforts should be made to enhance surveillance of antimicrobial resistance and healthcare-associated infections, including DFIs, through national and global surveillance systems. This includes monitoring antimicrobial resistance patterns, identifying emerging resistance trends, and implementing targeted interventions to prevent and control the spread of resistant pathogens.

## Conclusion

Diabetic foot infections constitute a multifaceted clinical condition, requiring a comprehensive approach to diagnosis, management, and prevention. Overall, in the current study, the predominant Gram-positive microbial species isolated in DFIs were Staphylococcus aureus, Enterococcus fecalis, Streptococcus pyogenes, Streptococcus agalactiae, and Staphylococcus epidermidis. Whereas the predominant Gram-negative included Escherichia coli, Klebsiella pneumoniae, Proteus mirabilis, and Pseudomonas aeruginosa. Risk factors for antimicrobial resistance in DFI encompass higher BMI, HbA1c, blood glucose levels and also ulcer characteristics, neuropathy, and vascular disease. MDT approach reduces DFIs with variation in team composition and podiatrists are crucial for DFI prevention and management. Addressing research limitations in DFI management through trials and collaborations is crucial. Future research on antibiotic resistance should focus on understanding mechanisms, developing agents, and stewardship. Recommendations include judicious prescribing, stewardship programs, R&D, and surveillance to combat resistance effectively and improve outcomes.

## Data availability statement

The original contributions presented in the study are included in the article/supplementary material. Further inquiries can be directed to the corresponding author.

## Author contributions

NN, SM, SN: Writing – original draft, Writing – review & editing, Validation, Investigation, Formal analysis. AJ, NL, NA: Writing – review & editing, Validation, Investigation, Formal analysis. DB, AA-I, KT: Writing - review & editing. SN, SM: Conceptualization, Project administration, Writing - original draft, Methodology, Supervision, Writing - review & editing. AC, YG, DB, AA-I: Writing - review & editing. SM, NL, NN: Contributed equally and share first authorship.
